# The Influence of Job Crafting on Nurses’ Intent to Stay: A Cross-Sectional Study

**DOI:** 10.3390/nursrep14040249

**Published:** 2024-11-11

**Authors:** Mª Carmen Rodríguez-García, Ángeles Ramos-Martínez, Celia Cruz-Cobo

**Affiliations:** 1 Department of Nursing, Physiotherapy and Medicine, Faculty of Health Sciences, University of Almeria, 04120 Almeria, Spain; mrg451@ual.es; 2 Research Group CTS-1127 Epidemiology and Public Health, University of Almeria, 04120 Almeria, Spain; 3 Distrito Sanitario Almería, Servicio Andaluz de Salud, Junta de Andalucía, 04120 Almeria, Spain; angelesrm9@gmail.com; 4 Department of Nursing and Physiotherapy, University of Cádiz, 11009 Cádiz, Spain; 5 Institute of Biomedical Research and Innovation of Cádiz (INiBICA), 11009 Cádiz, Spain; 6 Research Group on Nutrition, Molecular, Pathophysiological and Social Issues, University of Cádiz, 11009 Cádiz, Spain

**Keywords:** intent to stay, job crafting, nursing, retention

## Abstract

**Background/Objectives**: The increasing rates of nurse turnover pose significant challenges to healthcare systems, negatively impacting patient outcomes and increasing operational costs. Despite the recognized importance of retaining nursing staff, factors contributing to turnover intentions, such as job dissatisfaction and burnout, remain inadequately addressed. Developing job crafting skills among nurses can be a proactive strategy to mitigate these issues, leading to a more engaged and committed workforce. The aim of this study was to analyze nurses’ job crafting and its relationship with the intention to stay at their working hospitals or to leave the nursing profession. **Methods**: A cross-sectional, correlational study was conducted with a sample of 284 registered nurses using a self-reported online questionnaire with the standardized Spanish version of the Job Crafting Scale. Mann–Whitney U and Kruskal–Wallis nonparametric tests were used to determine statistically significant differences between two or more different groups for the job crafting variable, respectively. The Spearman correlation coefficient was calculated to explore the relationships between variables. **Results**: Mean scores obtained for the Job Crafting Scale indicated that nurses in the study had a high level of job crafting. Nurses with lower scores for the ‘Decreasing hindering job demands’ subscale had a significantly lower intention to stay at their workplace. Greater ‘Decreasing hindering job demands’ scores were significantly associated with a lower intention to leave the nursing profession. Lower nurses’ intention to leave the nursing profession was significantly associated with a greater intention to stay at hospitals. **Conclusions**: Improving ‘Decreasing hindering job demands’ job crafting skills to “decrease hindering job demands” through workload management, time management training, supportive supervision, resource availability, autonomy encouragement, promotion of team collaboration, and mental health support. It could lead to greater retention of nurses in their workplaces and in the nursing profession. Nursing managers and leaders should consider improving the job crafting skill “Decrease Hindering Job Demands” among nurses as a potential strategy for effective retention of nurses to address the challenges of the global nursing shortage.

## 1. Introduction

Job crafting in nursing is a concept that refers to the way in which nurses proactively and creatively modify or customize their tasks, interactions, or perception of their work to make it more meaningful and satisfying. It is a strategy that allows health professionals to adapt their role to their personal strengths, interests, and values, improving their well-being at work and reducing burnout [1]. Nurses play a fundamental role in the provision of care, carrying out their work immersed in a constantly changing and challenging healthcare context. They are responsible for providing complex patient care, managing organizational demands and maintaining professional relationships in fast-paced, high-pressure environments. Factors such as high workloads, staffing shortages, emotional exhaustion, and a perceived lack of support contribute to burnout and dissatisfaction, which can ultimately lead to high turnover rates and a growing intent to leave [1]. To ensure care delivery, nurses are expected to continuously adapt their professional practice to meet the demands based on available resources, which involves crafting their roles, managing workplace stressors, and prioritizing tasks that align with patient-centered care [2] This challenging context not only highlights the critical role of nursing workforces within healthcare systems but also creates an opportunity for nurses to engage in job crafting [3].

The concept of job crafting is based on employees’ response to organizational changes in the workplace [4]. Job crafting is defined as “the physical and cognitive changes individuals make in the task or relational boundaries of their work” [5] as well as “the self-initiated changes that employees make in their own job demands and job resources to attain and/or optimize their personal (work) goals”. From the perspective of job demands–resources (JD-R) theory, job crafting refers to proactive employee behaviors that aim to (1) increase structural job resources; (2) decrease hindering job demands; (3) increase social job resources; and (4) increase challenging job demands [6]. In the context of nursing practice, job crafting not only enhances individual job satisfaction but also improves patient care and organizational performance [7]. 

The literature shows that job crafting is a powerful strategy with which nurses can optimize their own functioning in the workplace, fostering the fit between person and organization [3]. Recent studies have shown that job crafting is positively associated with nurses’ happiness [7], well-being [8,9,10], personal empowerment, maintaining motivation, and quality of care [11]. However, most studies have focused on the positive relationship between job crafting and work engagement [12]. Researchers have provided evidence that developing job crafting of nurses leads to more committed staff with greater work engagement levels, improving teamwork and strengthening their relationships with colleagues [4] which might positively influence nurses’ intention to remain employed at their organizations [12] and in the nursing profession [13]. A previous study revealed that surgical residents with serious intentions of leaving exhibited lower levels of most job-crafting skills and work engagement, compared to those without such intentions [14]. Nevertheless, no studies on the relationship between job crafting and nurses’ intention to remain in their hospitals and profession have been found to date.

The relationship between job crafting and nurses’ intent to stay is highly relevant to clinical nursing practice. Given the increasing rates of nurse turnover, which negatively affects both patient outcomes and healthcare systems, exploring how job crafting can empower nurses to remain in their positions is critical. The impact of nurse turnover on nursing staff, patients, organizations, and society has been widely described in the literature. More specifically, it contributes to heightened stress and burnout among remaining nurses, reducing job satisfaction. For patients, turnover compromises patient safety and care continuity, leading to increased incidences of medical errors, hospital-acquired infections, and elevated mortality rates [1,2]. Also, it imposes substantial financial and operational burdens on healthcare organizations due to recruitment and training costs and exacerbates workforce shortages, ultimately undermining the capacity of healthcare systems to meet societal health. Retaining nursing staff within a hospital is important to eliminate the negative influence of voluntary turnover on the quality of care, organization costs [15], and nurse well-being. In the face of a global nurse shortage, it is now relevant to create strategies that contribute to the recruitment and retention of nurses [16]. 

Consequently, the following hypotheses will be tested:

**H1:**  *There is a significant relationship between nurses’ job crafting levels and their intention to remain at their current hospital*.

**H2:**  *Higher scores on the “Decreasing hindering job demands” subscale are significantly associated with a greater intention to stay at the organization*.

The aim of this study was to analyze nurses’ job crafting and its relationship with the intention to stay at their working hospitals or to leave the nursing profession.

## 2. Materials and Methods

### 2.1. Design

This is a cross-sectional, correlational, descriptive study. The “Strengthening the Reporting of Observational Studies in Epidemiology” (STROBE) checklist was used when reporting the study [17]. 

### 2.2. Participants

This study was conducted with a convenience sample of registered nurses working in hospitals throughout Spain. Eligible participants included registered nursing staff in medical, surgical, intensive care, emergency services, and operating theatres. The Raosoft sample size calculator was used to calculate the required sample size. Considering a population proportion of 50%, a confidence level of 95%, and a margin of error of 5%, the sample size was estimated to be 377. The inclusion criteria for nurses’ participation were as follows: (a) having a Bachelor of Science in Nursing; (b) working as a registered nurse; (c) working in hospital settings; (d) having at least one year of experience; and (e) signing informed consent. Nurses working in administrative or non-clinical roles without direct patient care responsibilities were excluded from the study.

### 2.3. Data Collection

Data were collected using an online questionnaire with the following sections: (1) study information and informed consent, (2) the Spanish version of the Job Crafting Scale, and (3) participants’ sociodemographic information. The questionnaire was designed on Google Forms and was estimated to take about 10–15 min to complete. A total of 380 registered nurses were invited to participate in the study via social media. A link to access the questionnaires was sent to potentially eligible subjects via social media, including WhatsApp. The link to the questionnaire was also posted on Facebook, specifically in nursing communities and private groups to encourage participation. Participants were provided with information regarding the purpose of the study, the anonymity of the collected data, and the voluntary nature of their participation. A total of 320 questionnaires were filled out, resulting in a response rate of 84%. The data collection lasted for one month, and, meanwhile, reminders were sent to encourage participation among individuals and increase the response rate. Incomplete questionnaires were discarded from the analysis. The final sample of the study consisted of 284 Spanish nurses.

### 2.4. Measurements

This study used the Spanish version of the Job Crafting Scale validated by Bakker et al. (2018) [3], which had acceptable Cronbach’s alpha values between 0.70 and 0.79. This scale consists of 21 items assessing four subscales: S1-Increasing structural job resources (five items); S2-Decreasing hindering job demands (six items); S3-Increasing social job resources (five items); and S4-Increasing challenging job demands (five items). Responses were given on a 5-point frequency scale (1 = never, 5 = very often) with higher scores indicating greater levels of job crafting. In this study, Cronbach’s alpha reliability coefficient of the scale was 0.787.

Sociodemographic variables of the participants (gender, age, level of education, years of nursing experience, hospital, work unit, working hours, intention to stay at their hospitals, and intention to leave the nursing profession) were collected. The variable work unit was classified into medical-surgical hospitalization and specialized units, including intensive care, emergency services, and operating theatres. Nurses’ intention to stay at their work hospitals was measured using the following question: ‘Do you plan to continue working as a nurse at the same hospital for the next few years?’. Nurses’ intention to leave the nursing profession was assessed as follows: ‘Have you considered leaving the nursing profession?’. Both questions were answered on an ordinal scale (yes—maybe—no).

### 2.5. Ethical Considerations

This study adhered to the ethical principles of the Declaration of Helsinki [15] and was carried out in accordance with the research proposal approved by the corresponding Institutional Review Board. Participants were informed about the purpose of the research and the voluntary nature of participation, reserving the right to withdraw from the study at any time. The authors ensured confidentiality and anonymity by assigning individual identification codes to participants and providing data in a consolidated form to prevent individual identification. Personal data were stored securely in encrypted digital files, and access was restricted only to the research team. Informed consent to participate was obtained from all of the participants in the study.

### 2.6. Data Analysis

Statistical analysis was performed using SPSS v28.0 for Windows. Percentages and frequencies were calculated for categorical variables. Central tendency and dispersion measures were obtained for quantitative variables. Normality was explored using the Kolmogorov–Smirnov test. The nonparametric Mann–Whitney U and Kruskal–Wallis H tests were used for comparisons between groups. The Spearman correlation coefficient was calculated for the relationship between job crafting mean scores and nurses’ intention to stay and intention to leave. In this study, the confidence interval was 95%, considering values of *p* < 0.05 to be significant.

### 2.7. Validity and Reliability

The data were collected using tested and validated instruments. In this study, the Cronbach’s alpha values of the Spanish version of the Job Crafting Scale were greater than the preferred 0.75 requirements of acceptability.

## 3. Results

### 3.1. Sociodemographic Characteristics of Study Participants

The sociodemographic characteristics of study participants are described in Table 1.

The study sample was primarily female (n = 246; 86.6%). Nurses ranged in age from 23 to 59 years, with an average age of 35 years (SD = 8.7) and had been working for a mean of 12 years (SD = 8.8). Regarding the level of education, most of the participants had a Bachelor of Science Degree in Nursing (n = 178; 62.7%). The distribution of the sample according to the work unit was similar with 50.7% (n = 144) and 49.3% (n = 140) of nurses working on medical-surgical hospitalization and specialized units, respectively. Although more than half of the nurses intended to stay at their working hospitals (n = 160; 56.3%) there were one in five nurses who were not sure (n = 68; 24%) and one in three nurses who intended to leave the nursing profession (n = 98; 34.5%).

### 3.2. Descriptive and Bivariate Analysis

The mean score of Job Crafting Scale was 3.52 ± 0.4, with ‘Increasing structural job resources’ having the highest mean subscale score (4.31 ± 0.4) and ‘Increasing social job resources’ having the lowest (2.98 ± 0.6). Means and standard deviations of job crafting items and subscales are shown in Table 2. 

Statistically significant differences were observed in the mean scores for the job crafting subscale ‘Decreasing hindering job demands’ regarding intention to stay at their working hospitals as determined using the Kruskal–Wallis test (*H*(2) = 7.008, *p* = 0.03). Registered nurses who scored lower on the ‘Decreasing hindering job demands’ subscale had a significantly lower intention to stay at their current workplace (Table 3). No statistically significant differences were observed for job crafting mean scores according to other variables and subscales.

### 3.3. Correlational Analysis

A Spearman correlation coefficient was calculated for the relationship between nursing job crafting and intention to stay at hospitals or intention to leave the nursing profession. A negative correlation was found (rs = −0.188, *p* = 0.02), indicating a significant relationship between the ‘Decreasing hindering job demands’ subscale and nurses’ intention to leave the nursing profession. Greater ‘Decreasing hindering job demands’ scores were significantly associated with a lower intention to leave the nursing profession. Furthermore, a negative correlation was also observed between nurses’ intention to leave the nursing profession and intention to stay at hospitals (rs = −0.255, *p* = 0.002), showing that nurses with lower intention to leave the nursing profession tend to have a greater intention to stay at their work hospitals.

## 4. Discussion

This study aimed to analyze nurses’ job crafting and its relationship with the intention to stay at their working hospitals or to leave the nursing profession. 

Mean scores obtained for the Job Crafting Scale indicated that nurses in the study had a high level of job crafting, similar to previous research where the ‘Increasing structural job resources’ was also the subscale that scored the highest [10,12,16]. ‘Increasing social job resources’ obtained the lowest score, as in the study of Baghdadi et al. (2021) [18], getting better scores in other studies where the lowest subscale scored by the nurses was ‘Decreasing hindering job demands’ [12,16].

Nurses with lower scores for the ‘Decreasing hindering job demands’ subscale had a significantly greater intention to leave their workplace. Furthermore, correlation analysis showed that the lower ‘Decreasing hindering job demands’ scores, the greater intention to leave the nursing profession, and a higher intention to leave the profession tends to a lower intention to stay at the work hospital. No previous research has been found to support this idea given the limited scientific production of job crafting in nursing to date. Thus, additional future studies are required to clear up this original finding.

In our country, a study aimed at analyzing the effects of job development activities of nursing home and elderly care employees on their perceived well-being and quality of care confirmed the effects of job development on quality of care. A positive linear relationship was found between job crafting and well-being with quality of care [11].

The results of this study reveal the importance of promoting the development of the ‘Decreasing hindering job demands’ job crafting skill to enhance nursing retention. However, this subscale is among the worst rated in the majority of studies published to date on job crafting in nursing [17,18], including in the present study. Nursing managers and leaders should be aware of these findings and focus all their efforts on improving this skill among nurses as a potential nurse retention strategy that might help address the global nursing staff shortage.

This study contributes to the literature, highlighting the significance of enhancing the job crafting levels of nurses and investing in their improvement as a potential strategy that might help to effectively manage one of the most critical organizational issues: nursing staff retention. Nursing managers and leaders should strive to enhance nurses’ job crafting by promoting a healthy work environment where nursing staff were provided with support and resources that allow them to effectively adjust the demands of their work and its intensity. Improving job crafting in nurses might lead not only to greater nurse work engagement [17], happiness [19], well-being, and quality of care [9], but might also prevent nursing staff from leaving their workplace and profession. Considering the expected nursing shortage in the coming years, the results of this study might prove useful and assist nursing managers in decision-making and the implementation of successful nursing staff-retention strategies. 

### Limitations and Future Research

The study had some limitations related to the sample and study design; thus, the results of this research should not be generalized. The sample was obtained through convenience sampling, which may limit the generalizability of the findings to the broader population of nurses. Future studies should consider employing random sampling methods to enhance the representativeness of the results. Additionally, the length of time nurses had worked in their current hospital or unit was not included as an eligibility criterion, though it could influence perceptions and experiences. Incorporating this variable in future research could provide a deeper understanding of how job tenure impacts the outcomes studied. The results should also not be understood as causal, as the nature of the study design allows for the establishment of associations between variables, but not causality. Considering the self-reported nature of the data, participants’ responses were guaranteed to be anonymous and confidential to prevent or reduce social desirability bias. In this study, the authors did not consider response rates in the sample size calculation. Addressing response rates in future studies is essential to ensure an adequate sample size and to minimize bias in the findings. The effectiveness of nurse retention strategies based on improving job crafting skills requires future research, as does the relationship between job crafting and nurses’ intention to stay or leave. It would be useful to carry out future studies in different settings and study samples in order to contrast the original findings of this study. Furthermore, studies confirming causality would provide nurse managers and leaders with more accurate information about the factors driving nursing retention. The strengths of the study include the use of a standardized and validated version of the Job Crafting Scale, which ensures that the results are comparable with other studies in the health care setting. This not only adds rigor to the research, but also facilitates the possibility of replicating the study in the future. Despite the limitations associated with convenience sampling, a significant sample of 284 nurses from different units and specialties was obtained. This diversity in the sample allows for a broader exploration of variations in job crafting and its relationship to tenure intention, thus enriching the analysis of the results.

## 5. Conclusions

This original study investigated nurses’ job crafting and its relationship with the intention to stay at their work hospitals and leave the nursing profession. In general, nurses participating in this study had good results for job crafting skills. Nurses with higher scores for the ‘Decreasing hindering job demands’ subscale showed a significantly greater intention to remain in their workplace. Greater ‘Decreasing hindering job demands’ tend to reduce nurses’ intention to leave nursing profession. In short, improving ‘Decreasing hindering job demands’ job crafting skill “decreases hindering job demands” through workload management, time management training, supportive supervision, resource availability, autonomy encouragement, promotion of team collaboration, and mental health support. It could lead to greater retention of nurses in their workplaces and in the nursing profession. Nursing managers and leaders should consider improving the job crafting skill “Decrease Hindering Job Demands” among nurses as a potential strategy for effective retention of nurses to address the challenges of the global nursing shortage.

## Figures and Tables

**Table 1:** Sociodemographic Characteristics of the Study Nurses (n = 284).

Study Variables	n (%) or Mean (SD)
Gender	
Male	38 (13.4)
Female	246 (86.6)
Age	34.96 (8.7)
Years of nursing experience	11.65 (8.8)
Level of education	
Bachelor of Science Degree in Nursing	178 (62.7)
Clinical Nurse Specialist	70 (24.6)
Master of Nursing Science	32 (11.3)
PhD	4 (1.4)
Work unit	
Medical-surgical hospitalization	144 (50.7)
Specialized units	140 (49.3)
Intention to stay at hospitals	
Yes	160 (56.3)
Maybe	68 (24)
No	56 (19.7)
Intention to leave nursing profession	
Yes	98 (34.5)
Maybe	32 (10.6)
No	156 (54.9)

**Table 2:** Job Crafting Scale Mean Scores.

Job Crafting Subscales and Items	Mean ± SD
S1. Increasing structural job resources	4.31 ± 0.45
Item 1. I try to develop my capabilities	4.48 ± 0.54
Item 2. I try to develop myself professionally	4.50 ± 0.58
Item 3. I try to learn new things at work	4.58 ± 0.53
Item 4. I make sure that I use my capacities to the fullest	4.45 ± 0.61
Item 5. I decide on my own how I do things	3.56 ± 1.11
S2. Decreasing hindering job demands	3.03 ± 0.89
Item 6. I make sure that my work is mentally less intense	3.32 ± 1.09
Item 7. I try to ensure that my work is emotionally less intense	3.37 ± 1.12
Item 8. I manage my work so that I try to minimize contact with people whose problems affect me emotionally	2.99 ± 1.19
Item 9. I organize my work so as to minimize contact with people whose expectations are unrealistic	3.23 ± 1.19
Item 10. I try to ensure that I do not have to make many difficult decisions at work	2.62 ± 1.20
Item 11. I organize my work in such a way to make sure that I do not have to concentrate for too long a period at once	2.66 ± 1.23
S3. Increasing social job resources	2.98 ± 0.69
Item 12. I ask my supervisor to coach me	2.19 ± 1.14
Item 13. I ask whether my supervisor is satisfied with my work	3.11 ± 1.30
Item 14. I look to my supervisor for inspiration capabilities	2.13 ± 1.19
Item 15. I ask others for feedback on my job performance	3.07 ± 1.28
Item 16. I ask colleagues for advice	4.40 ± 0.64
S4. Increasing challenging job demands	3.77 ± 0.71
Item 17. When an interesting project comes along, I offer myself proactively as project co-worker	3.99 ± 0.94
Item 18. If there are new developments, I am one of the first to learn about them and try them out	3.81 ± 0.98
Item 19. When there is not much to do at work, I see it as a chance to start new projects	3.60 ± 1.06
Item 20. I regularly take on extra tasks even though I do not receive extra salary for them	3.63 ± 1.15
Item 21. I try to make my work more challenging by examining the underlying relationships between aspects of my job	3.85 ± 0.91
Total	3.52 ± 0.44

**Table 3:** Nurses’ intent to stay at their work hospitals according to the ‘Decreasing hindering job demands’ Mean Scores.

	S2. Decreasing Hindering Job Demands				
Intention to Stay at Hospitals	N	(Mean ± SD)	χ^2^	df	*p*
No	28	2.61 ± 0.89	7.008	2	0.03 *
Maybe	34	3.21 ± 0.94			
Yes	80	3.09 ± 0.84			

* *p* < 0.05 derived from the Kruskal–Wallis test. Only statistically significant results are reported.

## Data Availability

The data are available upon request of the first author.

## Public Involvement Statement

There was no public involvement in any aspect of this research.

## Guidelines and Standards Statement

This manuscript was written following the STROBE guideline.
